# Pre-symptomatic autoimmunity in rheumatoid arthritis: when does the disease start?

**DOI:** 10.1007/s00281-017-0620-6

**Published:** 2017-03-23

**Authors:** Alexander Tracy, Christopher D. Buckley, Karim Raza

**Affiliations:** 1 Department of Rheumatology, Sandwell and West Birmingham Hospitals NHS Trust, Dudley Road, Birmingham, B18 7QH UK; 2 Rheumatology Research Group, Institute of Inflammation and Ageing, University of Birmingham, Birmingham, B15 2TT UK

**Keywords:** Rheumatoid arthritis, Autoimmunity, Inflammation, Autoantibodies

## Abstract

It is well recognised that a state of autoimmunity, in which immunological tolerance is broken, precedes the development of symptoms in the majority of patients with rheumatoid arthritis (RA). For individuals who will later develop seropositive disease, this manifests as autoantibodies directed against proteins that have undergone specific post-translational modifications. There is evidence that the induction of this autoantibody response occurs at peripheral extra-articular mucosal sites, such as the periodontium and lung. In addition to their utility as diagnostic markers, these autoantibodies may have a pathogenic role that helps localise disease to the synovium. Alongside the development of autoantibodies, other factors contributing to pre-symptomatic autoimmunity may include dysbiosis of the gastrointestinal tract, abnormal development of lymphoid tissue, and dysregulated autonomic and lipid-mediated anti-inflammatory signalling. These factors combine to skew the balance between pro-inflammatory and anti-inflammatory signalling in a manner that is permissive for the development of clinical arthritis. We present data to support the concept that the transitions from at-risk states to systemic autoimmunity and then to classifiable RA depend on multiple “switches”. However, further prospective studies are necessary to define the molecular basis of these switches and the specific features of pre-symptomatic autoimmunity, so that preventative treatments can be targeted to individuals at high risk for RA. In this review, we analyse mechanisms that may contribute to the development of autoimmunity in at-risk individuals and discuss the relationship between this pre-symptomatic state and subsequent development of RA.

## Introduction

Rheumatoid arthritis (RA) is a chronic inflammatory disease characterised by symmetrical peripheral polyarthritis that can lead to joint destruction and may be associated with extra-articular features. There has been increasing interest in studying its early stages with a view to modulating early pathogenic processes to prevent RA development [1].

Rheumatoid arthritis results from a complex interplay between genetic and environmental factors. Arguably, the presence of these factors represents the earliest stage in the pathogenesis of RA. Because the aetiology of RA is multifactorial, there has been some inconsistency in the terminology used to describe its earliest stages. However, EULAR recommendations have provided a standard system for describing phases leading up to the development of RA which we use throughout this review [2, 3] (Table 1). Phases A to C can be considered “at-risk” pre-symptomatic phases, while phases D and E represent symptomatic phases prior to the development of classifiable RA.

**Table 1:** Summary of EULAR terminology for phases of RA development (adapted from [3])

Phase	Definition
A	Genetic risk factors for RA
B	Environmental risk factors for RA
C	Systemic autoimmunity associated with RA
D	Symptoms without clinical arthritis
E	Unclassified arthritis
F	Rheumatoid arthritis

In a large proportion of patients, RA is associated with autoantibodies directed against post-translationally modified proteins/peptides including citrullinated (ACPAs), carbamylated (ACarPAs) and acetylated proteins/peptides (AAPAs) [4, 5] and/or autoantibodies against the Fc portion of IgG (rheumatoid factor, RF). Classically, ACPA-/RF-positive patients are defined as seropositive and are known to have a poor prognosis compared to seronegative patients [6]. It is now well established from retrospective studies that individuals may be seropositive, i.e. in phase C (EULAR definition), for many years before developing symptoms of inflammatory arthritis [7, 8]. More recently, these findings have been extended to include anti-carbamylated protein antibodies (ACarPAs) [9]. This represents important evidence for a pre-symptomatic state of systemic autoimmunity associated with RA.

An additional line of evidence for pre-symptomatic immunopathology comes from analysis of cytokine levels in blood samples from pre-symptomatic individuals who later developed RA. This has demonstrated increased expression of pro-inflammatory cytokines and chemokines compared to control subjects [10]. Findings such as these indicate that pathological immune processes significantly predate the onset of symptoms. Furthermore, there is magnetic resonance imaging (MRI) evidence of subclinical joint inflammation in a subset of individuals with arthralgia deemed suspicious for progression to RA [11]. Although histological analysis in pre-symptomatic phases has not always demonstrated synovitis, preliminary evidence is suggestive of subtle T-cell infiltration preceding the signs and symptoms of arthritis [12]. Therefore, even at the level of the joints, immune activation may occur before it can be detected clinically.

Based on these lines of evidence, it is now widely accepted that there is a prolonged state of autoimmunity that precedes symptom onset in RA. Using these immune characteristics, we may be able to develop strategies to stratify individuals for preventative interventions before development of clinical disease. It may then prove possible to modulate the aetiological processes in such a way that prevents progression to RA from phases A–E. However, a fuller understanding of pre-symptomatic autoimmunity is required before this is possible. Here, we review what is currently known on this subject and suggest how future research may enhance our understanding.

We refer predominantly to studies of individuals before the onset of symptoms of RA (phases A–C). However, some studies of early disease mechanisms in RA have included individuals in symptomatic at-risk disease stages (phases D and E). This literature is discussed where it helps to inform our understanding of very early disease mechanisms.

The majority of the literature cited in this review concerns seropositive RA. This is because the study of at-risk seronegative populations in the pre-symptomatic stage is much more challenging due to a lack of markers for risk of seronegative RA. This is a significant limitation of the literature, and as a result there has been very little work on early immune mechanisms of seronegative RA. However, it has been shown that a population of patients with seronegative arthralgia deemed prone to progression to RA have MRI evidence of subclinical synovitis [13]. This is analogous to evidence from seropositive at-risk subjects. Therefore, it would be beneficial to understand early disease mechanisms in seronegative RA, where the underlying immunopathology may be distinct from seropositive RA.

## Predisposition to rheumatoid arthritis: how has genetics informed our understanding of early immunopathology?

In many individuals, the first stage of the development of RA is the acquisition of genetic risk factors for the disease at conception. Arguably, this represents the “start” of the disease process and accordingly represents phase A in EULAR terminology. The study of genetic risk factors has contributed to our understanding of the initial immunopathology of RA, for example by emphasising the importance of T-cell activation in seropositive disease. The best-studied genetic risk factors for RA are specific variants at the HLA loci. The discovery that many RA-associated alleles within the *HLA-DRB1* gene share a conserved amino acid sequence led Gregersen and colleagues to propose the “shared epitope” (SE) hypothesis [14]. Interestingly, the presence of this amino acid sequence in the MHC class II molecule confers an increased risk of anti-citrullinated protein antibody (ACPA)-positive disease only [15]. More recent work has suggested that five amino acid positions explain most of the association between HLA alleles and seropositive RA [16]. Two of these are within the classical SE region. They are all located within peptide-binding grooves on MHC class I or II molecules, indicating that polymorphisms may have a functional impact on antigen presentation not only to CD4+ but also to CD8+ T-cells. Furthermore, outside the HLA loci, other genetic risk factors for RA involve genes that are implicated in T-cell activation e.g. *PTPN22*, *CTLA4* and *STAT4* [17].

Epidemiological investigations have evaluated the stage at which SE alleles impact the aetiology of seropositive RA. The presence of SE has been associated with ACPA positivity, high ACPA concentrations and reactivity of ACPAs to multiple, rather than single, autoantigens [18, 19]. SE alleles are associated more strongly with ACPA-positive RA (phase F) than with ACPA positivity in the absence of RA (phases C–E) [19]. This suggests that SE alleles may play a greater role in the switch from ACPA positivity to the development of RA than in the initial induction of ACPA positivity.

The interaction of SE alleles with environmental factors is controversial. A gene-environment interaction between SE alleles and smoking was found for seropositive RA in a large Swedish cohort [20] and in large Danish and Korean case-control studies [21, 22]. However, this was only partially replicated in three American cohorts [23]. There is also increasing interest in the interplay between SE status and other environmental factors, including omega-3 fatty acid consumption levels, on RA risk. There is evidence from case-control and prospective cohort studies suggesting that fish consumption is inversely correlated with risk of RA [24], which may be mediated by an effect on omega-3 fatty acid levels. In a case-control study, increased percentage of omega-3 fatty acids in red blood cells (RBCs) was inversely correlated with RF and ACPA positivity only in SE-positive participants [25]. The mechanism behind this is unknown, but the authors speculate that omega-3 fatty acids alter lipid rafts to change the conformation and expression of MHC class II molecules. This could have functional implications for autoantigen presentation [25].

While most genetic studies have focused on alleles associated with increased RA risk, there is an emerging role for protective alleles. For example, in North European cohorts, HLA-DRB1*13 has been associated with protection against the development of ACPA-positive RA, but not from the development of ACPA positivity in healthy subjects [26]. Furthermore, in participants with seropositive RA, HLA-DRB1*13 is associated with reduced ACPA levels and a narrower range of autoantigen recognition [26]. HLA-DRB1*13 and HLA-DRB1 SE alleles seem to have inverse but analogous effects, suggesting that they may influence the same pathological pathway. The authors hypothesise that HLA-DRB1*13 alleles act by mediating thymic deletion of a T-cell population that cross-reacts with microbial antigens and autoantigens [27]. Although the underlying mechanism is still unproven, these results suggest that the primary influence of HLA-DRB1 alleles is on the switch from phase C to F, i.e. the development of disease in the context of ACPA positivity.

Further insight into the genetic associations of RA comes from the finding that different genetic associations exist according to the profile of autoantigens recognised by ACPAs in a given individual [28]. One weakness of a significant portion of genetic studies is a failure to define these fine specificities, instead relying on anti-cyclic citrullinated peptide (anti-CCP) antibodies as a generic marker of ACPA status. A full understanding of the immunogenetics of RA will only be possible once the pathways leading to antibody reactivity to individual autoantigens have been defined.

Thus far, the genetic evidence discussed applies only to seropositive RA. A study of twin pairs with at least one RA twin has demonstrated that the heritability of ACPA-positive and ACPA-negative RA is comparable (68 and 66% respectively) [29]. However, HLA SE alleles explained significantly less of this heritability in ACPA-negative disease (2.4 versus 18%) [29]. As is the case for ACPA-positive disease, there is an association of ACPA-negative RA with variability at the HLA loci [30]. Recent work has identified distinct HLA alleles associated with seronegative RA [31], which suggests that the mechanisms underlying its HLA associations may differ slightly from seropositive disease.

Taken together, current genetic evidence implicates antigen presentation, either in the thymus or periphery, as a key step in the aetiology of RA. However, the development of symptoms depends on further non-heritable pathogenic processes. The remainder of this review focuses on the putative mechanisms that promote autoimmunity in genetically susceptible individuals.

## Loss of tolerance to “self” peptides after post-translational modification

In early RA, immune responses have been detected against a range of peptides/proteins that have undergone specific forms of post-translational modification. Autoantibodies directed against peptides/proteins that have undergone citrullination have been detected up to 9 years prior to the onset of symptoms [7, 8], suggesting that the loss of tolerance to citrullinated peptides/proteins is an early event in the development of RA. Retrospective analysis shows that epitope spreading follows loss of tolerance, such that the repertoire of peptides recognised by ACPAs expands as time to diagnosis decreases [32]. Furthermore, in a prospective cohort of subjects with seropositive arthralgia, recognition of multiple citrullinated peptides correlated with increased risk of developing arthritis during follow-up [33]. Alongside epitope spreading, the avidity of ACPAs increases from phase C until disease onset, when no further avidity maturation is observed [34]. Taken together, these results show that expansion of the immune response against citrullinated autoantigens is associated with progression from EULAR phase C to phase F.

A range of inflammatory processes may be responsible for the generation of these autoantigens. Citrullinated proteins have been identified in inflamed specimens from a range of tissue types, suggesting that citrullination may be induced non-specifically by local inflammation [35]. Various environmental factors may drive such processes, as discussed in detail in subsequent sections of this review. Smoking is one example of an environmental risk factor for RA that has been specifically linked with citrullination. For example, a study of specimens from bronchoalveolar lavage found that cells from smokers had higher expression of citrullinated proteins [36]. This was associated with increased expression of peptidylarginine deiminase isoform 2 (PAD2), which was likely responsible for smoking-induced citrullination [36]. One can therefore hypothesise that upregulation of PAD2 in respiratory mucosa causes local hypercitrullination and that this provides a source of autoantigens driving the development of ACPAs. This effect may explain the epidemiological observation that smoking is a risk factor for seropositive, but not seronegative, RA [37]. Interestingly, increased citrullination has not been observed in whole tissue specimens from bronchoscopic biopsy or lobectomy [36, 38], raising the possibility that the effect of cigarette smoke is restricted to the airspace-facing aspect of the alveolar compartment.

A specific component of the inflammatory response that may provide a source for citrullinated autoantigens is the formation of neutrophil extracellular traps (NETs). NETs are known to contain citrullinated proteins e.g. histone H1 and H4, presumably due to the action of neutrophil peptidylarginine deiminase isoform 4 (PAD4) [39]. Indeed, active PAD2 and PAD4 isoforms are released from NETotic neutrophils in vitro [40]. Citrullinated H4 from NETs is bound by antibodies from the serum of RA patients [41], showing that the products of NETosis can be targeted by ACPAs. Important work by Khandpur and colleagues showed that NETosis is enhanced in RA compared to osteoarthritis and that ACPAs can stimulate NET formation [42]. Therefore, by externalising citrullinated proteins, NETs may contribute to the induction and expansion of autoimmunity in a positive feedback loop [42].

Although the work discussed here primarily concerns citrullinated autoantigens, there is evidence of autoimmunity to peptides that have undergone other forms of post-translational modification. Firstly, a retrospective analysis of serum samples from a military cohort found that ACarPAs were associated with future diagnosis of RA [9]. Cross-sectional data have extended this finding to first-degree relatives of RA patients [43], and it would be informative to study in more detail the temporal relationship between ACarPA positivity and symptom onset. Secondly, in patients with early inflammatory arthritis, detection of antibodies against acetylated vimentin was associated with development of RA [5].

Therefore, at least three distinct post-translational modifications are associated with the early phases of RA development. It is not known whether the pathological processes that drive autoimmunity against these modified peptides are different or if the same underlying mechanism underlies the generation of all RA-associated antibodies. In addition to being a marker of RA, loss of tolerance to “self” peptides that have undergone post-translational modifications may contribute to the pathogenesis of RA.

## Evidence for a pathogenic role for RA-related autoantibodies

Epidemiological evidence has associated ACPA positivity with increased disease severity, particularly with radiographic progression [44] and all-cause mortality [45]. Such observations have stimulated investigation of the immunological and pathological effect of ACPAs in animal models of RA. Wigerblad and colleagues showed that administration of ACPA to mice resulted in IL-8-dependent pain-like behaviour without any evidence of inflammation [46]. This raises the possibility that ACPAs may be responsible for inducing arthralgia prior to the onset of joint inflammation i.e. that they may precipitate EULAR phase D. However, it is not clear how well the tests of mechanical/thermal hypersensitivity and assessment of pain-like behaviour used in this murine study recapitulate the symptoms experienced by RA patients, and of course not all patients with inflammatory arthralgia who eventually develop RA are ACPA-positive.

Further work builds on the finding that anti-mutated citrullinated vimentin autoantibodies induce differentiation of osteoclasts in vitro and stimulate bone-resorptive activity [47]. Krishnamurthy and colleagues demonstrated that polyclonal ACPAs isolated from RA patients induced osteoclastogenesis via a PAD-dependent IL-8-mediated autocrine mechanism [48]. Furthermore, loss of trabecular bone mineral density was observed when certain monoclonal ACPAs were administered to mice. These findings are consistent with imaging evidence from asymptomatic ACPA-positive human subjects showing reduced bone mineral density compared to ACPA-negative controls [49]. However, the dominant feature in humans is thinning and fenestration of the periarticular cortical bone, with thinning of the trabecular bone a milder phenomenon [49]. Therefore, although the evidence provided by Krishnamurthy et al. suggests that ACPAs could be responsible for bone loss in early RA, it is not clear that the murine process is equivalent to that in patients.

Thus far, the evidence presented suggests that the effect of ACPAs could account for some features of phases C–D of RA development. In addition, there is evidence that ACPAs could promote other aspects of immune activation that characterise RA. Firstly, immune complexes containing citrullinated fibrinogen and ACPAs from RA sera can induce tumour necrosis factor (TNF) secretion from macrophages [50]. This pro-inflammatory mechanism is mediated by the surface-expressed FcγIIa receptor. Further in vitro work has demonstrated that such immune complexes can also trigger macrophage TNF production by co-stimulation of toll-like receptor-4 (TLR-4) and Fcγ receptors [51]. Finally, purified ACPAs can induce NFκB activation and TNF production by monocytes in vitro by binding to the surface-expressed citrullinated Grp78 receptor [52]. Taken together, these experiments suggest multiple mechanisms by which ACPAs, either alone or as components of immune complexes, can stimulate macrophages to release pro-inflammatory cytokines implicated in RA pathogenesis.

Recent evidence suggests that glycosylation may regulate the pathogenicity of such autoantibodies. Pfeifle et al. have identified IL-23 and T_H_17 cells as decisive factors that promote the intrinsic inflammatory activity of autoantibodies in murine models of autoimmune arthritis [53]. A retrospective analysis of serum from asymptomatic ACPA-positive humans (phase C) demonstrated significantly reduced glycosylation of ACPAs prior to RA development [53]. This study defines an IL-23-T_H_17 cell-dependent pathway that could unmask a pre-existing breach in immunotolerance by downregulating β-galactoside α2,6-sialyltrasferase 1 activity in newly differentiating antibody-producing cells [53].

Therefore, there is some experimental support for a pathogenic role for ACPAs in early RA. ACPAs may be responsible for some of the symptoms and features of RA development and may play a role in driving the inflammatory process in seropositive disease. Some of this evidence derives from animal models of debatable face validity and from in vitro cell lines. In future, therapeutic manipulation of ACPAs during different phases of RA development in humans could provide valuable insight into their pathogenic role. This may be possible using anti-plasma cell therapies, tolerisation immunotherapy or the development of novel peptides to antagonise ACPAs [54–56]. If such strategies could prevent transition from at-risk phases to classifiable RA, this would provide stronger evidence that ACPAs are of pathogenic importance.

## The lung mucosa and the development of autoimmunity

As introduced above, smoking is linked with increased citrullination in bronchoalveolar lavage (BAL) samples [36]. In early untreated seropositive RA patients, increased local citrullination has been associated with parenchymal lung abnormalities on high-resolution computed tomography (HRCT) and with high levels of ACPAs in BAL fluid [57]. ACPA levels were higher in BAL fluid than in the serum, possibly reflecting local autoantibody production by lung tissue [57]. These findings raise the possibility that the respiratory mucosa could play a role in the early stages of RA-associated autoimmunity.

Support for this hypothesis derives from studies of individuals in earlier phases of RA development. For example, HRCT reveals airway abnormalities in seropositive individuals who have no evidence of inflammatory arthritis on clinical examination i.e. from EULAR phase C/D [58]. Important work by Willis and colleagues has demonstrated the presence of ACPAs/RF in both the sputum of patients with early RA and the sputum of healthy individuals with either RA family history or ACPA positivity [59]. In these at-risk subjects, the ratio of autoantibody to total immunoglobulin was higher in sputum than in serum [59]. Furthermore, in a subset of at-risk individuals, autoantibodies were present in sputum but absent in serum [59], indicating that autoantibodies are either generated or sequestered in lung tissue. Taken together, these results suggest either that the lungs are an early target for injury secondary to autoimmunity or that they are implicated in its development.

In this context, a case-control study identified an association between bronchiectasis, cystic fibrosis and anti-CCP positivity [60]. This is significant because it is consistent with the hypothesis that respiratory mucosal inflammation per se can induce ACPA production. However, prospective studies remain necessary to determine the temporal relationship between ACPA positivity and respiratory mucosal inflammation.

In addition, there is histological evidence of germinal centre formation and B-cell/plasma cell accumulation in bronchial biopsies from ACPA-positive patients with recent onset of RA [61]. This suggests that bronchial tissue is a site of antibody production, but does not itself directly imply generation of RA-related autoantibodies. It would be valuable to investigate the association between these histological features and the local levels of ACPAs. Replication of these results in pre-symptomatic ACPA-positive individuals, ideally with follow-up to identify development of classifiable RA, would strengthen the concept that lung-derived ACPAs play a pathogenic role in RA.

If autoantibodies are generated in the lung, it is plausible that they could cross-react with autoantigens in the synovium. Support for this comes from the proteomic identification of shared citrullinated peptides, in particular of citrullinated vimentin, from bronchial and synovial tissue in RA [62]. This finding is significant because citrullinated vimentin has previously been validated as a target for ACPAs [63]. Thus, inflammation-induced autoantibody production by lung tissue may directly contribute to the development of arthritis.

In summary, there is strong evidence for lung involvement early in RA and emerging indirect evidence for a role in generating RA-associated autoantibodies. Common citrullinated antigenic targets in the lung and joint tissue provide a plausible mechanism by which autoantibodies from the lung could promote joint disease. However, it is not yet known whether the processes described here actually contribute to, or are necessary for, the development of clinical arthritis. Finally, none of the evidence discussed here is applicable to the pathogenesis of seronegative RA.

## Periodontal tissue in pre-symptomatic rheumatoid arthritis

In addition to lung tissue, periodontal tissue has attracted substantial interest as a potential site for the initiation of autoimmunity associated with RA. This was stimulated by two observations. Firstly, the prevalence of RA is significantly increased in patients with periodontitis [64]. Secondly, in established RA, periodontitis has been correlated with positivity for ACPAs [65] and with increased disease activity [66]. These studies support a link between periodontitis, autoantibody status and RA, but do not establish a causal relationship.

A clue to a possible underlying mechanism for this association comes from studies of inflamed periodontal tissue. Inflamed periodontium expresses increased levels of PAD enzymes and citrullinated proteins [67], and anti-CCP antibodies have been detected in the gingival crevicular fluid of periodontitis patients [68]. This effect may be explained by the properties of the periodontal pathogen *Porphyromonas gingivalis*. *P. gingivalis* possesses a PAD enzyme that is capable of citrullinating fibrinogen and α-enolase, both of which are potential targets for ACPAs [69]. Indeed, in individuals with genetic risk factors for RA, antibodies to *P. gingivalis* have been associated with systemic RA-related autoantibodies [70, 71]. More recently, it has been shown that *P. gingivalis* PAD can undergo autocitrullination and that antibodies directed against this citrullinated form of PAD are more prevalent in RA patients compared to controls [72]. However, an antibody response to citrullinated PAD peptides was not found in individuals who later developed RA, suggesting that these antibodies are unlikely to play an aetiological role [73].

Taking the above together, it has been hypothesised that *P. gingivalis* is responsible for the induction of RA-associated autoimmunity in a subset of individuals. One prediction of this is that the prevalence of *P. gingivalis* would be higher in patients with new-onset RA than in healthy controls. However, this has not been shown [74], and one study found reduced prevalence of *P. gingivalis* in salivary samples from such patients [75]. There are a number of possible explanations for this finding. Firstly, *P. gingivalis* may not have a role in the aetiology of RA or the induction of ACPAs. Secondly, there may be additional factors other than the prevalence of *P. gingivalis* that modulate the host immune response and thus the likelihood of developing autoimmunity. It is also plausible that an immune response to *P. gingivalis* that cross-reacts with autoantigens could be effective at reducing its carriage. Prospective studies will help to clarify the relationship between *P. gingivalis* and RA-associated autoimmunity.

More broadly, metagenomic shotgun sequencing of salivary and dental samples has detected oral dysbiosis in RA patients [75]. However, because this study was cross-sectional in design, we cannot infer the chronology of these changes and the direction of causality is unclear. Again, a prospective study of individuals in phases A and B of RA development would shed some light on this.

Taken together, current evidence shows that the oral microbiome in RA is different to that observed in healthy controls. This may play a role in the development of autoimmunity, or it may simply be a consequence of the disease. Periodontal inflammation is associated with the presence of RA-related autoantibodies, but the aetiological significance of this is also unclear. Even if a causal link were to be established between periodontitis and RA, this would most likely account for the development of RA in only a subset of seropositive patients. Therefore, other tissues outside the oral cavity are likely to play a significant role.

## The intestinal microbiota in pre-symptomatic rheumatoid arthritis

The concept that the intestinal microbiota plays a role in the immunopathology of inflammatory arthritis is supported by experiments using the k/BxN mouse model. This mouse expresses both a T-cell receptor transgene and the MHC class II molecule Ag7 and develops inflammatory arthritis associated with anti-glucose-6-phosphate isomerase (anti-GPI) autoantibodies [76]. Wu and colleagues showed that the development of arthritis could be prevented by raising the mouse in a germ-free environment and could be restored by exposure to a single gut commensal bacterium *Candidatus Savagella* [77]. This process correlated with germinal centre formation, anti-GPI abundance and restoration of splenic T_H_17 populations in an IL-17-dependent manner [77]. In humans, certain bacteria, such as *Prevotella copri*, are more abundant in stool samples from new-onset RA patients than healthy controls [78]. Colonisation of mice with *P. copri* renders them more sensitive to chemically induced colitis [78], indicating that the bacterium has pro-inflammatory properties. Therefore, it is plausible that the composition of the intestinal microbiota could predispose to RA by inducing a pro-inflammatory state.

To further explore the role of intestinal dysbiosis in predisposition to arthritis, Maeda and colleagues inoculated faecal samples from early RA patients into germ-free arthritis-prone mice [79]. When treated with zymosan, these mice showed increased intestinal T_H_17 cells and more severe clinical arthritis scores [79]. A role for *P. copri* is suggested by the finding that dendritic cells exposed to the bacterium stimulated naïve T-cells from arthritis-prone mice to produce IL-17 in response to an autoantigen [79]. In addition, Pianta and colleagues identified subgroups of new-onset RA patients with differential IgA or IgG reactivity to an *HLA-DR*-presented peptide from *P. copri*. [80]. This reactivity appears to be specific to RA patients and correlates with T_H_17-weighted versus Th1-weighted immune responses. [80]. Intriguingly, patients with an IgA-dominant T_H_17-weighted response were more likely to display ACPA positivity than those with IgG-dominant Th1-weighted responses to *P copri*. Therefore, this study suggests that *P. copri* may influence ACPA production as well as the release of pro-inflammatory cytokines such as IL-17 by T-cells.

Although *P. copri* has attracted specific attention in this field, metagenomic shotgun sequencing has revealed a broader pattern of intestinal dysbiosis associated with RA [75]. In particular, *Haemophilus* species are depleted and *Lactobacillus* species over-represented in stool samples from RA patients [75]. It is not known whether this is a contributing factor to the development of RA or an effect of the disease. Notably, the prevalence of *Haemophilus* species negatively correlated with autoantibody levels, suggesting that the bacteria may play a protective immune-modulating role. Therefore, dysbiosis could predispose to RA due to both the overgrowth of pro-inflammatory species and the depletion of protective species that possess immunomodulatory functions. By a mechanism currently undetermined, treatment with a disease-modifying anti-rheumatic drug (DMARD) appears to partially restore the microbiome to a healthy state [75].

The hypothesis that intestinal dysbiosis contributes to RA pathogenesis has led to small-scale randomised studies of probiotic therapy for patients with established disease. These have demonstrated some clinical improvement with probiotic therapy, which is accompanied by a reduction in serum levels of certain pro-inflammatory cytokines [81, 82]. Such findings are consistent with the principle that the intestinal microbiota can influence RA by altering the balance of pro- and anti-inflammatory factors. Therapeutic approaches to the manipulation of the microbiota in individuals at risk of developing the disease would aid the evaluation of this hypothesis.

In summary, as in the oral cavity, the flora of the intestine is altered in RA compared to healthy individuals. Experimental evidence suggests that certain commensal bacteria can have systemic pro-inflammatory effects, and it is possible that others modulate the immune system to counteract this. RA-specific immune responses to commensal bacteria have been identified, suggesting they may have immunopathological significance. A major limitation of the studies discussed here is the use of samples from patients with classifiable disease, rather than subjects at risk of future RA. To determine the role that the intestinal microbiota may play in the aetiology of RA, prospective studies of individuals from EULAR phases A–C will be required.

## Dysregulation of anti-inflammatory and pro-resolution signalling pathways

Thus far, this review has largely discussed pro-inflammatory mechanisms that are thought to contribute to the development of pre-symptomatic autoimmunity in RA. However, there is increasing interest in anti-inflammatory pathways whose suppression may facilitate autoimmune disease. Here, we review evidence for dysregulation of anti-inflammatory lipid mediators and cholinergic signalling.

Firstly, the involvement of lipid signalling in RA development has emerged since early studies showed that omega-3 fatty acid supplementation could reduce symptoms in patients with classified RA [83]. This could potentially be explained by increased levels of anti-inflammatory eicosanoid-derived resolvins, as has been observed in plasma following omega-3 fatty acid supplementation [84]. Therefore, recent work has aimed to elucidate the role of resolving signalling in murine models of inflammatory arthritis.

For example, in the k/BxN model, administration of arthritogenic serum results in reduced local levels of pro-resolving lipid mediators in the arthritic joint [85]. Subsequently, D-series resolvins RvD1, RvD2 and RvD3 are upregulated during the resolution phase [85]. When resolution is delayed by a second serum challenge, levels of RvD3 are reduced suggesting that non-resolving inflammation is associated with dysregulated resolvin signalling [85]. In line with this hypothesis, serum levels of RvD3 are significantly reduced in RA patients compared to healthy controls [85]. Whether this is also true for at-risk individuals prior to RA development is not known. It would be informative to measure resolvin levels locally in human joints and to prospectively determine when this dysregulation occurs.

In the same k/BxN model, treatment with RvD1 reduced both clinical arthritis scores and leukocyte infiltration within the synovium [86]. Consistent with this, RvD1 and RvD4 isolated from human synovial fluid were both shown to reduce neutrophil migration to an IL-8 gradient in vitro [86]. Therefore, dysregulation of pro-resolving lipid mediators in RA could result in disinhibition of neutrophil chemotaxis, predisposing to chronic inflammation. This process may be especially relevant to the transition from early synovial inflammation to classifiable RA.

It has also been suggested that autonomic anti-inflammatory signalling is disrupted in RA. Koopman and colleagues recently demonstrated that resting heart rate (HR) was elevated prior to the onset of disease in individuals at risk for developing RA [87]. This is thought to reflect reduced vagal parasympathetic tone and was associated with increased risk for developing RA during follow-up [87]. A potential mechanism by which reduced parasympathetic activity could predispose to RA is suggested by the finding that individuals with higher resting HR had lower expression of the α7 nicotinic acetylcholine receptor (α7nAChR) on peripheral blood monocytes [87]. Activation of the α7nAChR has previously been shown to reduce secretion of pro-inflammatory cytokines by CD4^+^ T-cells and macrophages [88, 89]. Therefore, reduced activation of the cholinergic anti-inflammatory pathway could contribute to the pathogenesis of RA. However, the use of cardiac vagal tone as a surrogate marker for activity of the cholinergic anti-inflammatory pathway is controversial as the neuroanatomical basis for this pathway has not been elucidated [90]. Furthermore, it is possible that increased resting HR and reduced α7nAChR expression are consequences of early inflammatory processes rather than reflective of an underlying causal mechanism.

On the other hand, vagal stimulation in humans has been shown to reduce peripheral blood levels of TNF, IL-1β and IL-6 and to improve RA clinical disease severity scores in a non-placebo-controlled study [91]. Further randomised studies are required to confirm this finding. It would be interesting to study whether vagal stimulation could prevent the onset of RA in patients at risk for the disease. There are of course ethical barriers to performing such invasive procedures in healthy individuals, but these may be surmountable if their quantifiable risk of RA can be determined to be sufficiently high.

In summary, there is increasing focus on the contribution of dysregulated anti-inflammatory pathways to the pathogenesis of RA. Further work in human subjects is required to define these pathways and determine the nature of their dysregulation in pre-symptomatic individuals.

## Changes in lymphoid tissue preceding the onset of rheumatoid arthritis

Dysregulation of the balance between pro-inflammatory and anti-inflammatory cytokine production has also been observed in lymphoid tissue. The study of lymphoid tissue prior to the onset of RA has been facilitated by development of a needle-core inguinal lymph node biopsy technique [92, 93]. This allows lymph node tissue from individuals at risk of RA to be analysed using flow cytometry and transcriptional profiling. The first exploratory study to use these methods found an increased frequency of CD19+ B-cells in early arthritis compared to healthy controls, with a non-significant trend towards a similar increase in autoantibody-positive at-risk subjects [93].

This technique has subsequently been used in larger populations to characterise changes to CD4+ T-cells, CD8+ T-cells and innate lymphoid cells (ILCs). This demonstrated reduced frequencies of IL-4- and IL-10-secreting CD4+ T-cells in lymphoid tissue from at-risk seropositive individuals [94]. Such reduction in regulatory cytokine production may explain the observation that the pro-inflammatory T_H_1 phenotype was more common among CD4+ T-cells from early RA patients [94]. Furthermore, in at-risk subjects, there was a reduced frequency of double-positive IFNɣ/IL-10 and IL-17/IL-10 T-cells in the lymph node. This may represent impaired autoregulation of pro-inflammatory signalling by CD4+ T-cells in early phases of RA development [94]. Interestingly, there was reduced production of IFNɣ and IL-17 by CD4+ T-cells in an in vitro stimulation assay, possibly reflecting T-cell exhaustion secondary to sustained autoantigen exposure [94].

In the context of CD8+ T-cells, the assessment of inguinal lymph node tissue demonstrated increased populations of CD45RO+ memory cells and recently activated CD69+ cells in both at-risk and early RA patients [95]. Analogous to findings from CD4+ T-cells, in vitro stimulation assays were suggestive of CD8+ T-cell exhaustion [95]. Of interest, this study also demonstrated a decreased frequency of regulatory CD8+ IL-10+ T-cells in peripheral blood from early RA patients [95], reinforcing the role of immunoregulatory dysfunction in early disease.

Recently, needle-core lymph node biopsy has been used to investigate innate lymphoid cell (ILC) subsets in RA development [96]. Lymphoid tissue inducer (LTi) cells play an important role in the development of lymph nodes and were progressively reduced in seropositive at-risk individuals and RA patients compared to healthy controls [96]. The authors speculate that reduction in this cell count could reflect impaired lymph node remodelling. In addition, the IFNɣ-producing ILC1 population was increased in at-risk subjects and early RA patients, and the IL-17-producing ILC3 population was increased in early RA [96]. Both of these cell types are potentially pro-inflammatory, suggesting that early development of RA is characterised by a shift of the ILC population towards an activated rather than homeostatic phenotype. However, the functional implications of these changes in vivo are unclear.

The studies discussed here provide new insights into changes within lymphoid tissue associated with phases C–E of RA development. Taken together, they suggest a dysregulation of lymphocyte function and an early bias towards pro-inflammatory phenotypes. However, this work is limited by practical difficulties in obtaining lymph node biopsies from large numbers of patients and healthy individuals. Longitudinal follow-up would be useful to determine whether particular features of lymphoid tissue are associated with future progression to RA.

## The transition from pre-symptomatic autoimmunity to the development of joint symptoms

This review has mostly focused on mechanisms outside joint tissue which may contribute to the development of pre-symptomatic systemic autoimmunity. For the transition from phases A–C to phases D–F, there must be additional mechanisms by which systemic autoimmunity begins to affect the joints. A number of possibilities have been suggested.

Firstly, it has been demonstrated that identical antigenic targets exist at mucosal sites where tolerance might be broken and at synovial tissues [62]. Therefore, antibodies directed against mucosal neoantigens could cross-react with synovial peptides and thus promote arthritis. This mechanism does not explain the delay between initial ACPA positivity and symptom onset, although there may be a small subgroup of patients in whom symptoms develop without this delay. In this putative population, induction of cross-reactive autoantibodies could cause rapid symptom development. Furthermore, IL-23/T_H_17-mediated downregulation of antibody glycosylation may represent a delayed event that induces pathogenicity of pre-existing cross-reactive autoantibodies [53].

Alternatively, epitope spreading could account for a process by which tolerance is lost to one autoantigen at a mucosal site, leading to the development of autoantibodies to a slightly different antigen at the synovium. Prior to the onset of RA, epitope spreading and avidity maturation occur, so that ACPAs progressively recognise more fine specificities and bind to autoantigens with higher avidity [34, 97]. However, only minimal further epitope spreading and avidity maturation is observed after diagnosis of RA [34, 97]. We can speculate that this represents a threshold effect, with clinical disease developing only once the ACPA response has matured sufficiently.

Thirdly, the existence of circulating immune complexes containing citrullinated autoantigens could provide a mechanism by which autoantibodies cause joint symptoms. Immune complexes containing citrullinated fibrinogen co-localise with complement component C3 in the rheumatoid synovium and are capable of stimulating macrophages via multiple receptors [50, 51, 98]. The preferential localisation of immune complexes to the joint could be explained by the specificities of constituent autoantibodies and potentially by local features of the synovial vasculature [99].

Furthermore, many processes discussed in this review likely combine to alter the balance between pro-inflammatory and anti-inflammatory signals in a manner that is permissive for arthritis development. Factors that contribute to this may include overgrowth of pro-inflammatory bacteria in the gastrointestinal tract, dysregulated autonomic signalling, aberrant anti-inflammatory lipid pathways and a bias towards T_H_1 and T_H_17 differentiation. These immunological mechanisms are likely to interact with additional environmental factors, for example trauma [100], to promote the development of early joint symptoms.

An important prospective study by de Hair and colleagues used MRI and mini-arthroscopic synovial biopsy to define features of the synovium in seropositive individuals without clinical arthritis [12]. In most individuals, there was no significant subclinical synovitis and no clear association between the presence of inflammatory cells and subsequent development of arthritis [12]. This implies that subclinical inflammation is not a characteristic feature of at-risk phases of RA development prior to phase E, although there was a non-significant trend towards an association between synovial CD3+ T-cell numbers and later progression to arthritis [12]. One caveat to this conclusion is that it depends on results from biopsies of the knee, which is not typically one of the first joints to be affected in RA.

On the other hand, de Hair and colleagues found that the presence of synovial CD8+ T-cells was associated with the presence of specific ACPAs and with the total number of ACPAs detected [12]. Thus, there may be a role for CD8+ T-cells directed against citrullinated peptides in the joint, but there was no statistically significant association between the presence of these cells and development of RA. The function of this synovial CD8+ T-cell population is an important topic for future research. This may provide new insight into mechanisms underlying the development of arthralgia and then clinically apparent synovitis in seropositive individuals.

## Conclusion

Here, we outline the mechanisms that are likely to contribute to pre-symptomatic autoimmunity associated with RA. In seropositive disease, there is clear evidence for autoimmunity directed against “self” peptides that have undergone specific forms of post-translational modification. This phenomenon may be triggered by non-specific local inflammatory processes outside the joints, for example in the respiratory mucosa. There is also some evidence for a role for microbial molecular mimicry and cross-reactive antibody responses.

In addition, this review considers multiple mechanisms by which pro- and anti-inflammatory factors become dysregulated, which may promote both the development of autoimmunity and subsequent progression to clinical disease. These include dysbiosis of the gastrointestinal tract, altered lipid signalling pathways and aberrant T-cell differentiation in lymphoid tissue.

A major limitation of the literature is its focus on autoantibodies with limited understanding of their pathogenic role. These autoantibodies have great utility as diagnostic markers and for easily defining cohorts of individuals at risk for RA. While there is some evidence indicating that ACPAs may contribute to disease development, their role in RA pathogenesis is not clear. The importance of the autoantibody response will not be fully understood until it can be manipulated in humans for prophylactic and therapeutic studies.

Because these autoantibodies are so useful in defining cohorts of “at-risk” individuals, seronegative RA has been relatively understudied. The majority of research discussed here is applicable only to seropositive disease, and there is very little understanding of the immunopathology underlying seronegative RA. This is important because seronegative disease is likely to be distinct in its aetiology.

The question posed by this review, “when does the disease start?” has no single simple answer. The EULAR terminology for stages of RA development forms a useful conceptual framework around which we have structured our thinking. Accordingly, we propose a model in which multiple “switches” are required for the development of RA, analogous to Knudson’s multiple-hit hypothesis of oncogenesis [101]. The first stage is genetic susceptibility, which is then augmented by environmental risk factors that act to promote inflammation. These may include smoking, trauma and microbiological factors.

Arguably, the acquisition of hereditary risk factors at conception represents the “start” of RA pathogenesis, but this is not conceptually useful because the disease is multifactorial. Alternatively, given that many individuals in phases A–C never develop RA, we might consider the onset of symptoms to be the start of disease. However, by this point, the underlying immunopathology may be well-established. Therefore, there is no individual stage that we define as the start of disease, and it is better considered as a prolonged process characterised by multiple superimposed switches. Nevertheless, this is an extremely important question for future scientific and clinical research. In particular, if we better understand the mechanisms of pre-symptomatic autoimmunity and the switches that trigger the transition between different phases of RA development, we may be able to modulate these for clinical benefit. This raises the possibility of future prophylactic interventions for individuals who can be identified as high risk for RA.
